# Organelle acidification negatively regulates vacuole membrane fusion *in vivo*

**DOI:** 10.1038/srep29045

**Published:** 2016-07-01

**Authors:** Yann Desfougères, Stefano Vavassori, Maria Rompf, Ruta Gerasimaite, Andreas Mayer

**Affiliations:** 1 Department of Biochemistry, University of Lausanne, Ch. des Boveresses 155, 1066 Epalinges, Switzerland

## Abstract

The V-ATPase is a proton pump consisting of a membrane-integral V_0_ sector and a peripheral V_1_ sector, which carries the ATPase activity. *In vitro* studies of yeast vacuole fusion and evidence from worms, flies, zebrafish and mice suggested that V_0_ interacts with the SNARE machinery for membrane fusion, that it promotes the induction of hemifusion and that this activity requires physical presence of V_0_ rather than its proton pump activity. A recent *in vivo* study in yeast has challenged these interpretations, concluding that fusion required solely lumenal acidification but not the V_0_ sector itself. Here, we identify the reasons for this discrepancy and reconcile it. We find that acute pharmacological or physiological inhibition of V-ATPase pump activity de-acidifies the vacuole lumen in living yeast cells within minutes. Time-lapse microscopy revealed that de-acidification induces vacuole fusion rather than inhibiting it. Cells expressing mutated V_0_ subunits that maintain vacuolar acidity were blocked in this fusion. Thus, proton pump activity of the V-ATPase negatively regulates vacuole fusion *in vivo*. Vacuole fusion *in vivo* does, however, require physical presence of a fusion-competent V_0_ sector.

Vacuoles of yeast cells have served as a model to study important aspects of membrane fusion in eukaryotic cells. Key elements of the vacuolar fusion apparatus have been identified by genetic screens^1^,^2^,^3^,^4^,^5^,^6^ and an *in vitro* system reconstituting fusion of purified vacuoles served to elucidate important mechanistic aspects^7^,^8^,^9^. Reconstitution experiments with pure proteins provided reduced fusion systems allowing to study the contributions of vacuolar SNAREs, the tether complex HOPS and the Rab-GTPase Ypt7 separately^10^,^11^,^12^,^13^,^14^. Studies on the fusion of isolated yeast vacuoles indicated a physical role of the membrane-integral sector of the V-ATPase, V_0_, in vacuole fusion that could be separated from its function in proton pumping. They also showed that V_0_ interacts with vacuolar SNARE proteins^7^,^9^,^15^,^16^.

Data from the endo-lysosomal system and at the plasma membrane in several other systems confirmed this finding and suggested that stimulation of fusion processes by V_0_ is a widespread phenomenon. Unbiased genetic screens identified V_0_ alleles causing defects in the fusion of synaptic vesicles, multivesicular bodies and phagosomes^15^,^17^,^18^,^19^,^20^. Targeted approaches in vertebrates implicated V_0_ in secretion of insulin, neurotransmitters and catecholamines^21^,^22^,^23^,^24^. All these studies provide evidence that the observed fusion defects depend on the physical presence of V_0_ and on its interaction with SNAREs. In none of the cases could they be explained by a loss of V-ATPase proton pump function. A recent study addressing the role of V_0_ for yeast vacuole fusion *in vivo* challenged the conclusions from the studies mentioned above and suggested that fusion required only acidity of the organelle lumen but not the V_0_ sector^25^.

Care must be taken to distinguish acidification-dependent and purely physical roles of the V-ATPase in every trafficking reaction under study. In yeast, a genetic separation of the functions of V_0_ in proton translocation and vacuole fusion was achieved by a random mutagenesis screen which produced point mutants in the V_0_ proteolipid subunits c (Vma3), c’ (Vma11) and c” (Vma16) and by protein fusions between c and c” subunits, which largely maintain proton pump activity but show strong reductions in fusion activity *in vitro*^15^,^26^. The substitutions that inactivate vacuole fusion are all in the transmembrane domains of the different V_0_ proteolipids, i.e. at sites where the affected amino acids could not directly interact with any V_1_ subunit. Therefore, their effect was explained by an altered conformation of V_0_ that allows proton pump activity but does not support the activity of V_0_ in vacuole fusion.

In living yeast cells, vacuoles fuse during organelle transmission in mitosis. Vacuoles produce tubulo-vesicular inheritance structures that are transmitted to daughter cells and fuse there^27^. A direct, real-time assay of this fusion event *in vivo* poses challenges because the process is slow and poorly synchronized. Therefore, vacuole morphology at steady state is frequently used as a substitute. This is based on the assumption that, upon a strong block of vacuole fusion, vacuolar fragments should accumulate over time, leading to an overall fragmented appearance of the vacuolar compartment. In line with this, vacuolar fusion problems often correlate with a fragmented vacuolar phenotype^2^,^6^. Vacuole inheritance and fusion occur also during mating. Upon mating, the zygote divides and its first diploid daughter cell receives vacuolar membranes from both mating partners, which fuse in the daughter^27^. Vacuolar material also flows from the bud to the mothers but this process has not been investigated in detail. Yeast mating has recently been used to analyze the *in vivo* requirements of vacuole fusion in daughters. The results led the authors to conclude that vacuole fusion *in vivo* required only vacuolar acidity and not the physical properties of V_0_. These results challenged *in vitro* studies from several laboratories that had suggested that the fusion of isolated vacuoles *in vitro* requires the physical presence of V_0_ and not V-ATPase pump function^7^,^9^,^15^,^16^,^28^,^29^,^30^.

In order to address this contradiction between these *in vivo* and *in vitro* observations we investigated the behaviour of vacuoles upon loss of the vacuolar proton pump function *in vivo*. We relied on video microscopy to analyze the morphological changes that follow the acute inactivation of V-ATPase proton pump activity and the resulting loss of vacuolar acidity. Our observations support, in good agreement with all *in vitro* results, the physical and pump-independent role of the V_0_ sector to promote vacuole fusion and suggest that the proton gradient counteracts vacuole fusion *in vivo*. We resolve the discrepancy between our observations and those of a previous *in vivo* study^25^.

## Results

**Figure 1:**
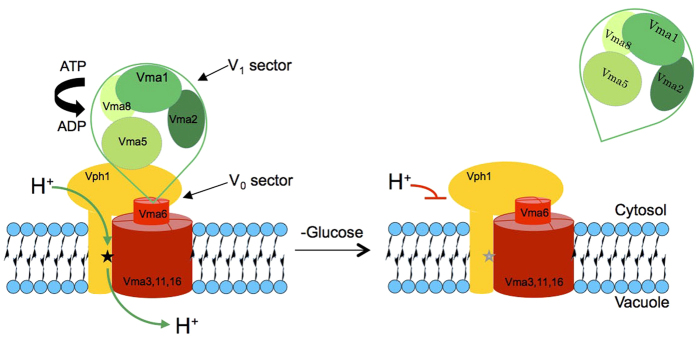
Organisation of V-ATPase and its glucose-dependent dissociation. V-ATPase is composed of a peripheral sector V_1_ (green), which carries the ATPase activity, and the proton-conducting, membrane-integral V_0_ sector (red and yellow). Only the V_1_ and V_0_ sector subunits used in this work are indicated. The black star in the V_0_ a-subunit Vph1 represents the residue R735. Glucose withdrawal releases V_1_ from V_0_ and renders it soluble in the cytosol. Proton conductance by V_0_ is blocked in this state.

V_0_ reversibly associates with the peripheral V_1_ sector in order to form the V-ATPase holo-enzyme, which is active as a proton pump (Fig. 1). V_1_ carries the ATPase activity. In absence of V_1_, V_0_ cannot conduct protons but it assembles normally and is targeted to vacuoles^31^,^32^ (Suppl. Fig. 1). Thus, if a fusion defect is due to a loss of proton pump activity it should become equally visible in a V_1_ mutant, which eliminates pump activity as effectively as a V_0_ mutant but leaves V_0_ intact. If V_0_ deletion shows additional defects compared to a V_1_ deletion, those cannot be accounted for by defects in proton pump activity, but they are consistent with a physical role of V_0_.

### Vacuoles in pump-deficient V-ATPase V_1_ mutants fuse into a single organelle

**Figure 2:**
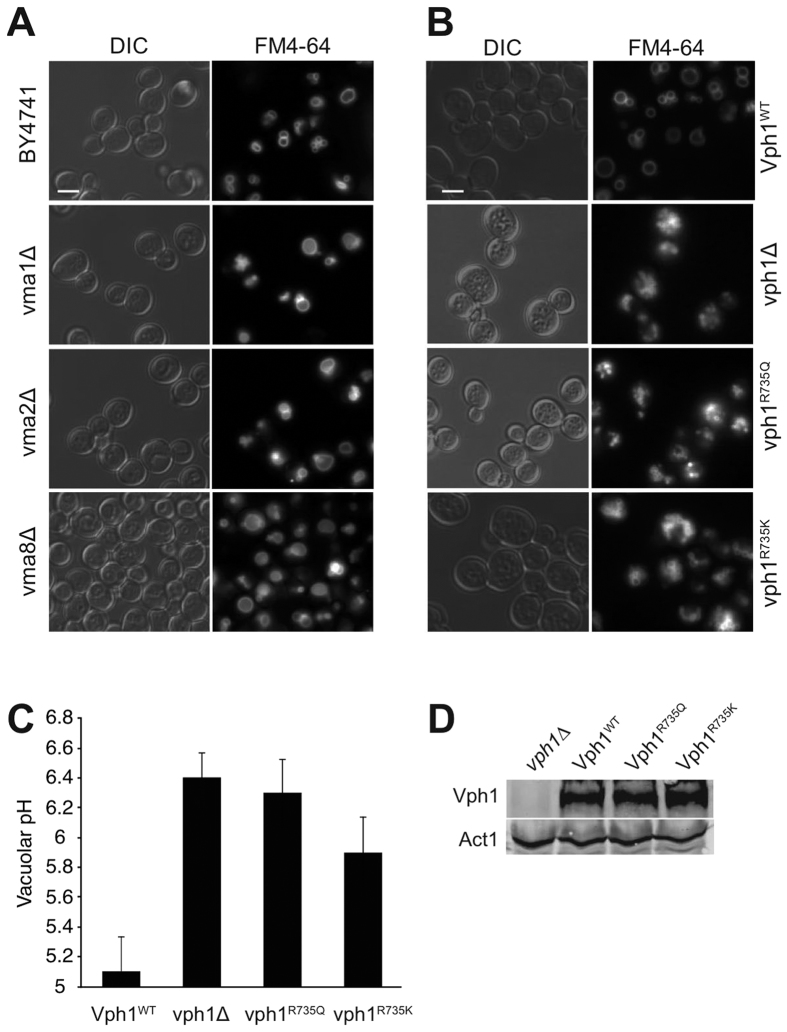
The morphology produced by vph1 mutations is atypical among mutants lacking V-ATPase pump activity. BY4741 cells were grown in buffered media to logarithmic phase, stained with FM4-64, and visualized by fluorescence microscopy and DIC. Scale bar: 5 μm. (**A**) Cells with deletions in various V_1_ subunits. (**B**) *vph1Δ* cells reconstituted with plasmids expressing the indicated alleles. (**C**) Cells from panel B were loaded with BCECF-AM and vacuolar pH was determined by fluorescence spectroscopy. Error bars represent Standard Deviation. (n = 9–22) (**D**) Total cell extracts of the cells from panel B were analyzed by SDS-PAGE and Western blotting against Vph1 and actin (Act1).

Vacuole fusion problems are often correlated with vacuolar fragmentation *in vivo*^6^,^33^,^34^. Using this simple *in vivo* criterion for vacuolar fusion activity we assessed vacuolar structure in a series of V_1_ and V_0_ mutants. We used several mutants in which one of the eight V_1_ subunits had been deleted, which abolishes proton pump function but leaves the V_0_ sector assembled and un-modified^35^. Under logarithmic growth, V_1_ deletion mutants showed a large vacuole whereas the isogenic wildtype (BY4741) showed several smaller vacuoles per cell (Fig. 2A). By contrast, mutants lacking the vacuolar isoform of the V_0_ a-subunit Vph1 showed strong vacuolar fragmentation, as did cells expressing a *vph1*^*R735Q*^ or a *vph1*^*R735K*^ allele (Fig. 2B,D). Since the *vph1*^*R735Q*^ allele abolishes V-ATPase pump activity but maintains an assembled V_0_ sector, the presence of fragmented vacuoles had been taken as an indication that vacuole fusion requires V-ATPase pump activity^25^ rather than SNARE-dependent conformational changes of V_0_^15^. A strong caveat for this interpretation is that *vph1*^*R735Q*^ interferes with the physiological dissociation of V_1_ from V_0_, which is triggered by glucose depletion^36^. Since R735 is in one of the transmembrane regions of the a-subunit, it could not directly interact with any V_1_ subunit. Its effect on V_0_/V_1_ association is therefore best explained by an influence on the conformation of V_0_, which is quite flexible and dependent on Vph1^37^,^38^. This is consistent with the phenotype of the *vph1*^*R735K*^ mutant, which also shows fragmented vacuoles. However, in contrast to *vph1*^*R735Q*^, *vph1*^*R735K*^ does support growth in alkaline media^39^, suggesting that V-ATPase remains at least partially functional as a pump. We found the vacuolar pH in *vph1*^*R735K*^ cells to be 5.9 whereas it was 6.3 in *vph1*^*R735Q*^ mutants, 6.4 in *vph1Δ* cells and 5.1 in wildtype cells (Fig. 2C). Coonrod *et al*. had concluded that the fusion defect of *vph1Δ* vacuoles *in vivo* could be rescued by expressing an artificial proton pump that lowered vacuolar pH by less than 0.2 pH units, from 6.6 to 6.4. If this were true, *vph1*^*R735K*^ vacuoles should be sufficiently acidified to fuse into a large, intact organelle. That this is not observed is consistent with an influence of R735 substitutions on vacuole fusion via the conformation or interactions of V_0_.

### Acute inactivation of V-ATPase induces vacuole fusion *in vivo*

**Figure 3:**
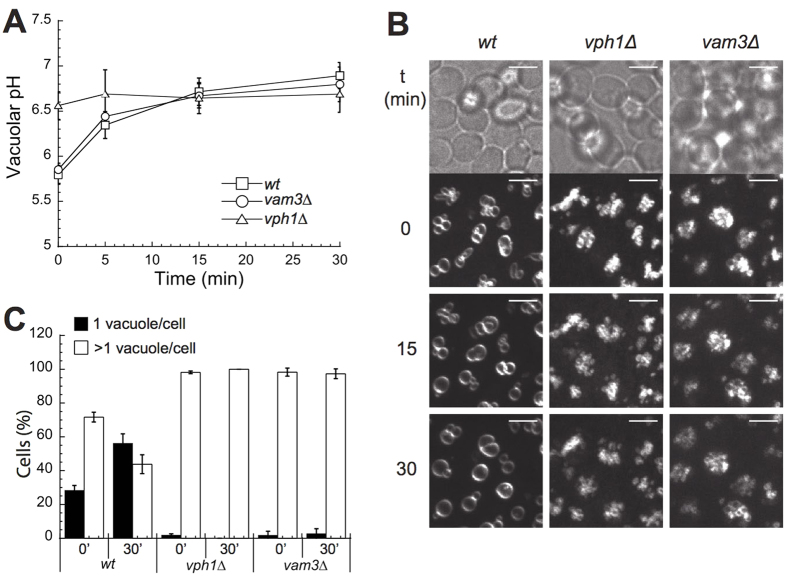
Vacuole fusion after addition of the pumping inhibitor concanamycin A. (**A**) Vacuoles of the indicated BY4741 cells were loaded with BCECF-AM and vacuolar pH was measured before and at different time points after addition of concanamycin (**A**). (**B**) Logarithmically grown cells stained with the vacuolar dye FM4-64 were immobilized in chamber slides as described and overlaid with 200 μl SC media. 200 μl media containing 4 μM concanamycin A (from a 200 μM stock in DMSO) were added to each well (final concentration 2 μM). At the indicated time points, image stacks were acquired using a confocal spinning disc setup. Maximum projections of the stacks are shown. Scale bar: 5 μm. (**C**) Quantification of the experiments in panel B. A number of 60–170 cells were classified according to the number of vacuoles per cell (n = 4).

The fusion of vacuoles into a single large vacuole in V_1_ mutants, which lack V-ATPase pump activity entirely, suggested that the lack of pump activity might actually favor vacuole fusion *in vivo*. We addressed this hypothesis by time-lapse fluorescence microscopy following acute inactivation of the V-ATPase. Concanamycin A is a highly potent and specific cell-permeable inhibitor of V-ATPases that allows to rapidly inactivate this proton pump in living cells. We added Concanamycin A to logarithmically growing cells in which vacuoles had been stained by the vital dye FM4-64. Cells were immobilized in chamber slides so that we could follow the changes in vacuolar structure by video-microscopy over the next 30 min. In parallel, we measured changes in vacuolar pH over time. Under these conditions, wildtype cells showed a vacuolar pH of around 5.6 and several smaller vacuoles (Fig. 3). Addition of concanamycin A led to a progressive increase in vacuolar pH to 6.5 after 15 min and to 6.7 after 30 min (Fig. 3A). In good correlation to the loss of vacuolar acidity, the vacuoles in many cells began to fuse after 10 min and this process was completed for essentially all cells in the population within 30 min (Fig. 3B,C). We also tested cells lacking the vacuolar SNARE Vam3, a core protein of the vacuolar fusion machinery. *vam3Δ* cells showed a vacuolar pH of 5.8. Addition of concanamycin A neutralized their pH to 6.7 but the vacuoles of *vam3Δ* cells did not fuse (Fig. 3B,C).

**Figure 4:**
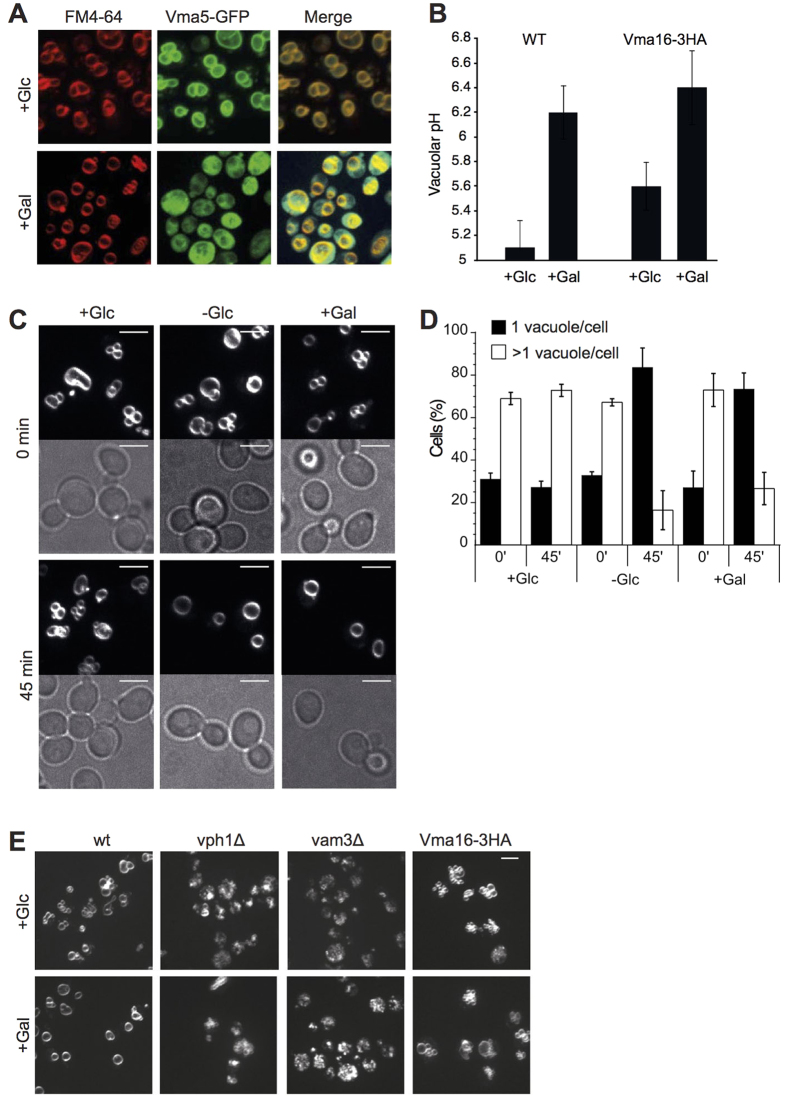
Vacuoles coalesce upon shift to media without glucose. (**A**) Effect of glucose withdrawal on V-ATPase assembly. Logarithmically growing BY4741 cells expressing Vma5-GFP were labeled with FM4-64, shifted from glucose media (+Glc) to media containing galactose (+Gal) and analyzed by fluorescence microscopy immediately or after 45 min incubation at 30 °C. (**B**) Vacuolar de-acidification in response to withdrawal of glucose. The indicated cells were grown as in (**A**) and vacuolar pH was measured before and after shift to galactose. Error bars represent Standard Deviation (n = 6). (**C**) Logarithmically growing cells, labeled with FM4-64, were shifted from glucose media to media containing no glucose (-Glc), galactose (+Gal) or glucose (+Glc, control). Cells were analyzed by fluorescence microscopy immediately or after 45 min of incubation at 30 °C. Scale bars: 5 μm. (**D**) Quantification of experiments as shown in (**C**). 50–220 cells were classified according to the number of vacuoles per cell (n = 3). (**E**) Response of mutants to glucose withdrawal. The indicated strains were subjected to a shift from glucose to galactose-containing media as in C and analyzed by fluorescence microscopy. Only FM4-64 fluorescence is shown.

As an alternative, non-pharmacological approach to inactivate vacuolar proton pump activity we used acute glucose withdrawal. In case of glucose shortage, yeast cells rapidly dissociate their V_1_ sectors from V_0_ in order to inactivate their V-ATPases, leading to de-acidification of the vacuole^40^,^41^,^42^. Switching from the preferred substrate glucose to other carbon sources, such as galactose or amino acids, requires an adaptation phase, which entails a transient bottleneck in carbon metabolism and downregulation of V-ATPase activity. We assessed the changes of vacuolar structure and vacuolar pH under these conditions. Cells growing on buffered medium with glucose (SC+Glc) were shifted to either SC-Glc (without glucose), SC+Gal (with galactose) or SC+Glc, and analyzed by fluorescence microscopy (Fig. 4). Withdrawal of glucose, or its replacement by galactose, led to a displacement of the V_1_ subunit Vma5-GFP from vacuoles into the cytosol (Fig. 4A). Vacuoles of wildtype cells were de-acidified from pH 5.1 to pH 6.2 (Fig. 4B) and fused into a single large organelle (Fig. 4C,D). The transition from several clustered vacuoles to a single fused vacuole was usually direct, suggesting that over this short time period re-fission of vacuoles hardly occurs (Fig. 4C, Suppl. Movies 1 and 2). Glucose withdrawal did not induce vacuoles fusion in cells lacking Vph1 or the SNARE Vam3 (Fig. 4E). We also tested V_0_ mutants that have a functional proton pump but are deficient for vacuole fusion *in vitro*, such as a strain in which the V_0_ proteolipids Vma16 and Vma3 are expressed as a single fusion protein (*vma16-3HA*)^26^. Upon shift from glucose to galactose, *vma16-3HA* cells showed vacuolar de-acidification from pH 5.6 to pH 6.4 (Fig. 4B). However, their vacuoles fused only poorly (Fig. 4E), consistently with the low vacuolar fusion activity that this mutant had shown *in vitro* (Strasser *et al*.^15^). Thus, both pharmacological and metabolic reduction of V-ATPase proton pump function induce vacuole fusion. This fusion depends on vacuolar SANREs and requires physical properties of V_0_ that are perturbed in the pump-active mutant *vma16-3HA*.

### Vacuole fusion in zygotes is independent of the vacuolar R-SNARE Nyv1

**Figure 5:**
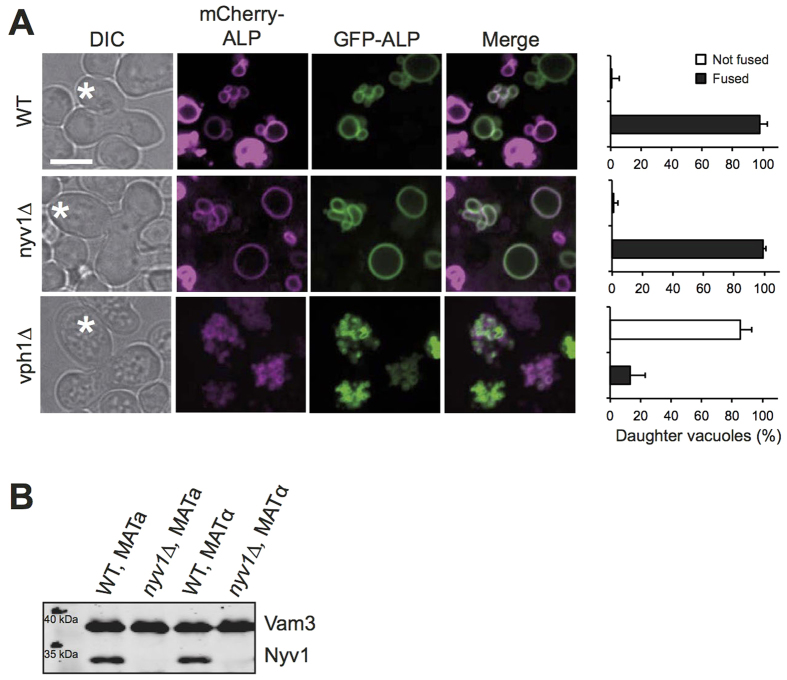
Vacuole fusion upon mating. (**A**) Plasmids expressing the PHO8 gene (ALP) fused to mCherry or GFP were introduced into isogenic a- and α-strains of BY4741 cells or the indicated nyv1Δ or vph1Δ mutants. a- and α-cells were grown over night in HC-Leu medium. In the morning, the cultures were diluted to OD_600nm_ = 0.2, the respective a and α cells were mixed and incubated for 4–6 h at 30 °C. Upon emergence of the first diploid daughters (labeled by an asterisk), the cells were analyzed by spinning disc fluorescence microscopy. The frequency of daughter cells showing complete co-localization of mCherry and GFP in their vacuoles was determined from 90 cells (n = 3). (**B**) Vacuoles were isolated from the wildtype and nyv1Δ cells used in A. Their proteins were analyzed by SDS-PAGE and Western blotting against Vam3 and Nyv1.

A recent *in vivo* study had concluded that vacuolar acidity rather than a physical contribution of V_0_ is necessary and sufficient to induce vacuole fusion^25^. In order to understand the qualitative differences between the *in vivo* observations in this study and ours, we revisited the *in vivo* approaches taken by Coonrod *et al*. These authors relied on yeast mating to study vacuole fusion. Upon mating, a yeast zygote divides and its first diploid daughter cell receives vacuolar vesicles from both mating partners, which fuse in the daughter^27^. Enzymatic and microscopic assays had been set up as readouts for this fusion event. The mating partners were genetically modified such that one partner carried a plasmid expressing a pro-alkaline phosphatase (pro-ALP) in its vacuoles and the other partner expressed the vacuolar maturase Pep4. Alternatively, the vacuoles of the two mating partners were labeled by differentially colored fluorescent proteins. Vacuole fusion in the daughter results in co-localization of the two fluorescent proteins and it gives Pep4 access to the pro-ALP, which is thereby converted into its mature form. We repeated the mating experiments described by Coonrod *et al*., using their fluorescently labeled mating partners that carried the vacuolar alkaline phosphatase ALP labeled with mCherry or GFP, respectively. We observed efficient co-localization of both fluorophores in daughters of wildtype zygotes but not in those of *vph1Δ* zygotes (Fig. 5A), confirming their observations^25^. However, both fluorophores efficiently co-localized also in daughters lacking the vacuolar R-SNARE Nyv1, suggesting that this protein is not required for vacuole fusion during mating. The lack of Nyv1 in these cells had been verified by Western blotting (Fig. 5B). This result is consistent with a previous observation that mixing of vacuole content during mating is Nyv1-independent^1^. It contradicts Coonrod *et al*., who reported that vacuole fusion during mating was Nyv1-dependent^25^. However, these authors had assessed the effect of Nyv1 only via the maturation of pro-ALP by Pep4.

### Vacuole fusion during mating cannot be assayed by maturation of vacuolar pro-enzymes

**Figure 6:**
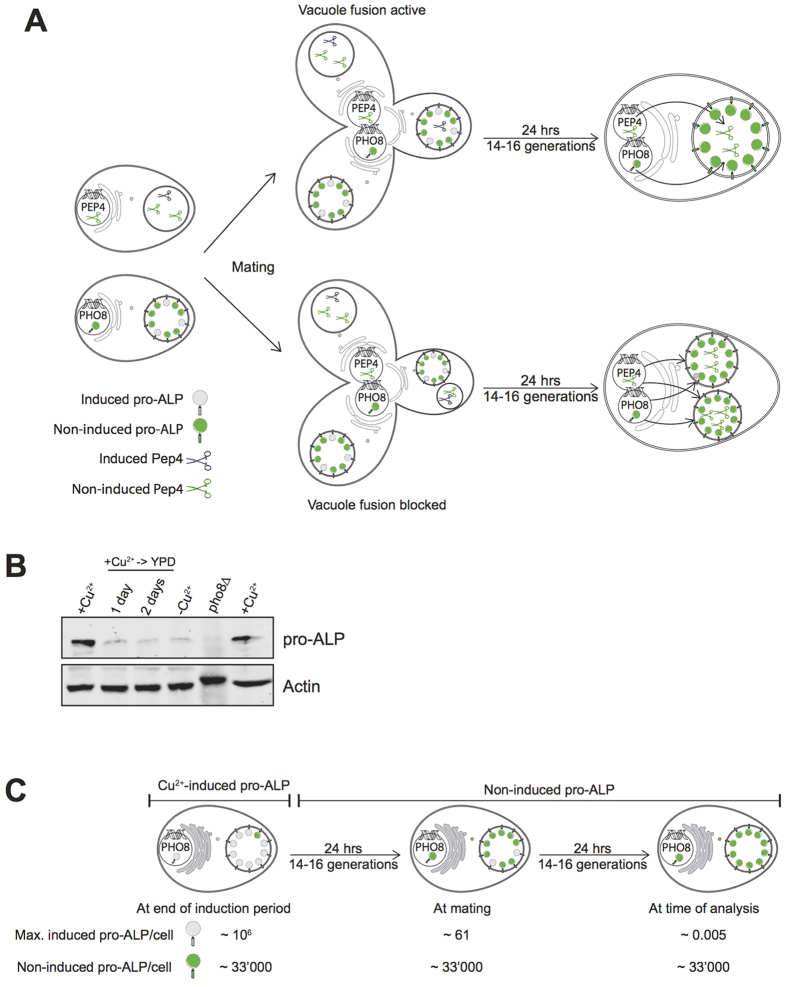
Limitations of the *in vivo* fusion assay employed by Coonrod *et al*. (**A**) The figure illustrates the mating procedure and the potential fate of proteins expressed from plasmids carrying the PHO8 gene (producing pro-ALP) or the PEP4 gene (producing its maturase proteinase (**A**) under control of the inducible CUP1 promotor. Induced pro-ALP is indicated by grey circles, pro-ALP resulting from constitutive, non-induced background expression is indicated by green circles. Induced Pep4 is indicated by grey scissors, Pep4 resulting from non-induced background expression by black scissors. For discussion, see main text. (**B**) Dilution of induced pro-ALP over time. KEBY136 cells^25^ expressing PHO8 from the CUP1 promotor were grown on SC-URA plus 50 μM CuCl_2_ for 24 h and then shifted to YPD without copper. Aliquots of cells were withdrawn before and 24 and 48 h after the shift and analyzed by SDS-PAGE and Western blotting against ALP and actin. Samples for background controls were taken from cells that had never been induced by copper and from pho8Δ cells that lacked the PHO8-expressing plasmid (KEBY136). (**C**) Conservative estimation of the abundance of induced pro-ALP relative to pro-ALP resulting from non-induced expression of PHO8 from the CUP1 promotor, resulting from the published mating assay for vacuole fusion^25^. The first 24h period represents a period of cultivation of the cells used to chase pro-ALP from the biosynthetic pathway. The second 24 h culture period that follows mating is used to select for diploid offspring.

We tried to understand the discrepancies between our microscopic observations and the results from the enzymatic Pep4/ALP assay published by Coonrod *et al*. by analyzing their assay procedure. The enzymatic assay relies on the assumption that the pro-ALP activated by vacuole fusion in the diploid offspring stems from the haploid mating partner and was not produced during the diploid phase, between mating and the final analysis of ALP activity (Fig. 6A). This is critical because mating takes many hours and the cytosols and genomes of the mating partners are already mixed in the diploids long before the daughter cell emerges. As a consequence, the diploids can express both pro-ALP and PEP4 and deliver them to the nascent daughter that serves to analyze vacuole fusion. Coonrod *et al*. tried to circumvent this problem by expressing pro-ALP and PEP4 from the copper-inducible CUP1 promoter, which allows to accumulate these proteins prior to mating and repress further expression during and after mating. However, since mating is slow and not quantitative, the mating products had to be selected before ALP maturation was measured by Western blotting. This selection via complementary auxotrophic markers requires a 24 h culture period that allows only zygotes to grow. The CUP1 promoters are repressed during this period. However, the diploids divide, leading to tremendous dilution of the pre-accumulated pro-ALP and Pep4.

A further important constraint is that, like every inducible promoter, the CUP1 promoter does not provide an absolutely tight on/off switch. Non-induced expression from the CUP1 promotor occurs at 1/10–1/30 of the rate achieved upon copper induction^25^,^43^,^44^. This creates a low but constitutive background expression of pro-ALP and Pep4. Both proteins resulting from this background expression are delivered to all vacuoles in the diploids. But the assay can only work if the pro-ALP induced and accumulated before mating gives rise to the major fraction of ALP observed after mating, i.e. if it remains significantly above the level of constitutive background expression. We verified this by Western blotting of ALP (Fig. 6B). Induction by copper (Cu^2+^) generated a strong signal, as published^25^. Within 24 h after transfer to medium without copper, this signal declined to a background level of approximately 8% that remained constant during futher growth of the cells for up to 92 h. It was equal to the signal observed before induction (-Cu^2+^). This signal was absent in extracts from cells lacking pro-ALP (*pho8Δ*). Thus, cell division dilutes the pre-accumulated pro-ALP to background levels in less than 24 h. Since the mating assay includes two growth periods for the diploids of 24 h each, both of which occur after the CUP1- pro-ALP construct has been shut down, the ALP signal measured after mating is dominated by the constitutively expressed ALP that was produced in the diploid.

This conclusion is strengthened by a conservative estimation of the fate of the induced pro-ALP during the maturation-based mating assay (Fig. 6C). The generation time of yeast in rich media is around 90–100 minutes, i.e. a cultivation period of 24 h allows 14 to 16 divisions. After the cessation of copper induction, the cells grow in rich media for 24 h, they are mated for 3–5 h and then cultivated for yet another 24 h in order to select for the diploids^25^. During both 24 h incubations, the cells divide 14–16 times. This dilutes the pro-ALP that has been produced during the preceding induction period by a factor of 2^28^ to 2^32^, i.e. more than 200 million-fold. The most abundantly expressed proteins in yeast are present at <10^6^ copies per cell^21^. Even if we assume that induced expression of the CUP1-pro-ALP construct were exceptionally efficient and reached this level, the dilution of the initially induced 10^6^ pro-ALP molecules by division would leave 10^6^/2^14^ < 61 molecules/cell after the first 24 h growth period and 10^6^/2^28^ < 0.005 molecules/cell after the selection for diploids. Furthermore, the half-life of ALP is <9 h in yeast^45^. Protein degradation over 48 h will hence diminish ALP further, bringing its maximal abundance to 61/2^2^ < 16 molecules per cell after 24 h and to 0.005/2^5^ < 1.5^*^10^−4^ at the time of analysis at 48 h. At the same time, non-induced, constitutive background expression from the CUP1 promoter occurs in every newly produced cell, creating a constant background level of around 1/30 of the inducible level, equivalent to 1/30 * 10^6^ = 33'000 molecules per cell. It exceeds the level of pro-ALP accumulated prior to mating by a factor of more than 6*10^6^. Thus, the pool of pro-ALP that is finally assayed stems entirely from constitutive background expression. Its major fraction, 1-(16/33'000) ≫ 99.9%, will have been produced after mating and will have been delivered to the vacuoles by biosynthetic transport, independently of vacuole-vacuole fusion (Fig. 6A,B). The same applies to the maturation enzyme Pep4. Here, the effect of background level expression is even more severe because few Pep4 molecules biosynthetically delivered to a vacuole should suffice to activate vacuolar hydrolases and the pro-ALP contained in it.

Thus, both our experimental results and theoretical considerations indicate that the mating assay as conceived by Coonrod *et al*. cannot report vacuole fusion in the zygotes because its signal is dominated by biosynthetic trafficking of constitutively expressed pro-ALP to the vacuoles. This might explain how these authors could observe defective ALP maturation with various mutants, such as *vam3Δ*, which lacks the vacuolar Q_a_-SNARE that is necessary for biosynthetic delivery to vacuoles^46^. However, we cannot rationalize how Coonrod et al. could have observed Nyv1-dependence for pro-ALP maturation because biosynthetic delivery routes towards vacuoles are Nyv1-independent^47^ and also vacuole fusion in zygotes is Nyv1-independent, as observed by us and by others^1^.

### The pyrophosphate-driven proton pump GFP-AVP1 alters vacuole structure *in vivo*

**Figure 7:**
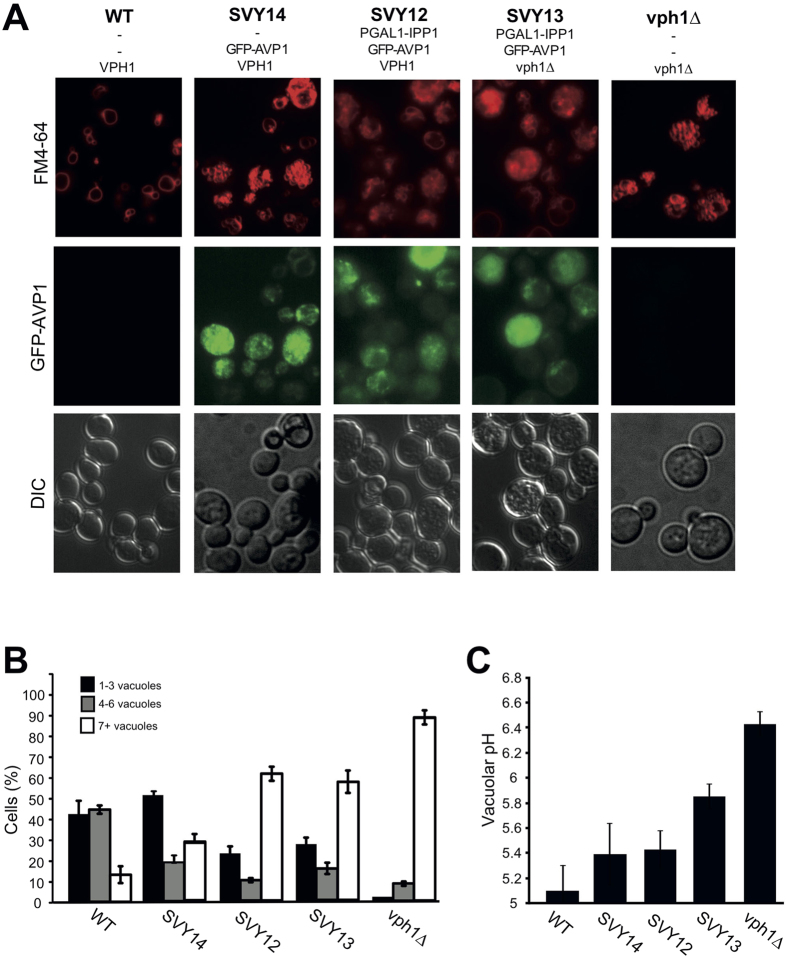
Effect of GFP-AVP1 expression on vacuolar structure and pH. (**A**) Vacuolar morphology. V-ATPase proficient cells (BY4741) or *vph1Δ* cells, and cells expressing GPF-AVP1 in these backgrounds (SVY12, SVY13, SVY14), were grown at pH 5.5 to logarithmic phase, stained with FM4-64, and analyzed by spinning disc fluorescence microscopy. (**B**) Classification of the cells shown in panel A according to the number of vacuoles per cell. 200–400 cells were analyzed per strain (n = 3–5). (**C**) Vacuolar pH was measured for the cells used in (**A**). (n = 7–9).

An ideal way of proving that the only contribution of V-ATPase to vacuole fusion is its proton pump function would be to replace V-ATPase by an unrelated proton pump, such as a proton-pumping pyrophosphatase from *Arabidopsis thaliana* (AVP1). In an attempt to re-establish vacuolar acidity independently of the V-ATPase, Coonrod *et al*. expressed GFP-AVP1 in *vph1Δ* cells. AVP1 pump activity depends on hydrolysis of pyrophosphate, a metabolite that is toxic to yeast cells. Pyrophosphate accumulates to levels supporting AVP1 activity only upon downregulation of the endogenous essential pyrophosphatase Ipp1^48^. Coonrod *et al*. reported that they had created *ipp1Δ* mutants expressing GFP-AVP1 from a high-copy plasmid^48^. Coonrod *et al*. were unable to send us these *ipp1Δ* cells expressing GFP-AVP1 (LGY 253, LGY254) that provide the central argument for their conclusions. We had independently tried to create such a strain and could not obtain viable cells, presumably because Ipp1 is essential and the detoxification of pyrophosphate by GFP-AVP1 alone is insufficient for cell survival. This matches the experience of Perez-Castineira *et al*., who did not analyze the effects of GFP-AVP1-expression in *ipp1Δ* cells but used mutants in which IPP1 was expressed from a regulatable GAL promotor^48^,^49^,^50^. Even in the repressed state, residual expression from the Gal promotor appears to produce a minimal amount of Ipp1 that remains necessary for survival in presence of GFP-AVP1. Therefore, we used these strains with Gal-regulated IPP1 to revisit the effects of GFP-AVP1 on vacuole morphology. The GFP-AVP1 plasmid was, however, identical to the one used by Coonrod *et al*. While *vph1Δ* cells showed fragmented vacuoles, expression of GFP-AVP1 in these cells yielded a heterogeneous population, with 30% of the cells showing 1–3 large vacuoles and >50% maintaining fragmented vacuolar morphology (strain SVY13; Fig. 7A,B). This matches previous observations, which had been taken as evidence that the re-acidification of *vph1Δ* vacuoles by GFP-AVP1 had rescued vacuole fusion^25^. As a control, we expressed GFP-AVP1 in cells proficient for VPH1 and for IPP1, which maintained vacuolar acidification via V-ATPase. Expression of GFP-AVP1 changed vacuolar morphology in these VPH1-proficient and IPP1-proficient cells, increasing the frequency of cells with very numerous, highly fragmented vacuoles from <10% to 30% (strain SVY14; Fig. 7A,B). Additional downregulation of IPP1 (strain SVY12) exacerbated this effect, yielding a vacuolar morphology quite similar to the distribution observed in *vph1Δ* expressing AVP1. Thus, expression of GFP-AVP1 and downregulation of IPP1 strongly perturbs vacuolar morphology, even in V-ATPase-proficient cells. This renders it problematic to ascribe the segregation of the *vph1Δ* population into cells with fragmented and non-fragmented vacuoles to a rescue of vacuolar acidity and fusion by GFP-AVP1.

Next, we tested the effects of GFP-AVP1 on vacuolar pH. As reported^25^, GFP-AVP1 expression in *vph1Δ* rescued vacuolar acidity partially, from pH 6.4 to pH 5.8, whereas the vacuolar pH in the wildtype was 5.1 under the same conditions (Fig. 7C). Seeking to explain the re-appearance of larger vacuoles in the *vph1Δ*/GFP-AVP1 strain as a consequence of the re-activation of vacuolar fusion by AVP1-dependent vacuole acidification, two interpretations are possible: First, an acidification to pH of 5.8 might already support vacuole fusion. If this were true, the effects of numerous V_0_ mutants that are deficient for vacuole fusion *in vivo* and *in vitro*^15^ could not be explained by a lack of vacuolar acidification. Our measurements of such mutants yielded for example pH = 5.6 for *vma16-3HA* (Fig. 4B). Nevertheless, this mutant maintains fragmented vacuoles *in vivo* (see above) and shows drastically lower fusion activity *in vitro*^15^. Thus, if pH 5.8 were necessary and sufficient for vacuole fusion, the fusion defect of this mutant supports a physical role of V_0_ in vacuole fusion *in vivo* that is independent of its role in proton pumping. The second possibility is that the expression of AVP1 is heterogeneous, with half of the cells expressing GFP-AVP1 insufficiently, showing higher-than-average pH and keeping fragmented vacuoles, and half of them expressing it strongly, allowing lower-than-average vacuolar pH and fusion. The latter hypothesis had been put forward^25^ but it had not been experimentally verified. We analyzed the intensity of GFP-AVP1 expression by fluorescence microscopy. Cells presenting a large vacuole usually showed barely detectable levels of GFP-AVP1 whereas cells showing high GFP-AVP1 signals typically had fragmented vacuoles (Suppl. Figs 2 and 3).This observation contradicts the assumption that the subpopulation of *vph1Δ*/GFP-AVP1 cells that shows a large vacuole does so because it expresses AVP1 more highly than the average and has more acidic vacuoles. Also in the wildtype background, the presence of a large vacuole was inversely correlated with the GFP-AVP1 signal (Suppl. Fig. 4).

### Expression of GFP-AVP1 interferes with vacuole fragmentation

**Figure 8:**
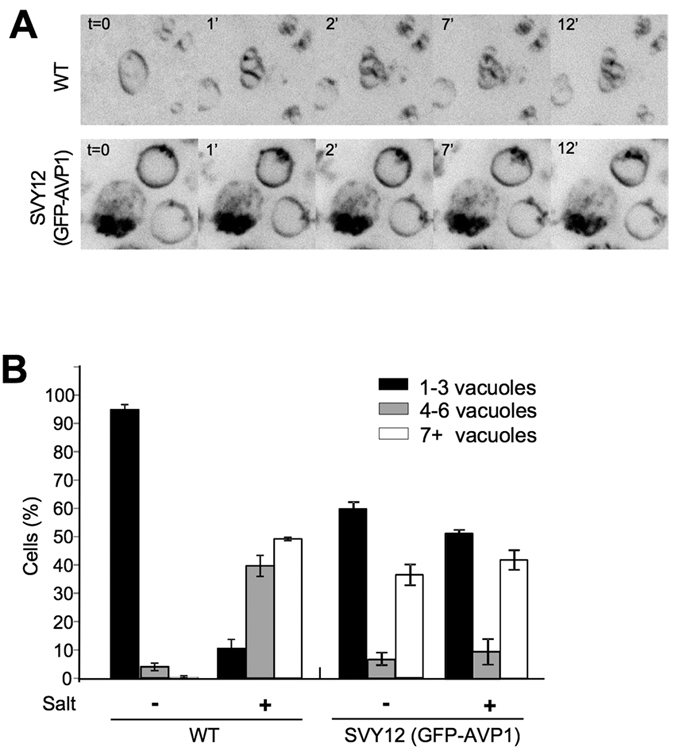
Effect of GFP-AVP1 on vacuolar fission activity. (**A**) AVP1 expression impairs vacuole fragmentation. Wildtype cells (BY4741) and cells expressing GFP-AVP1 (SVY12) were grown logarithmically in SC medium and labeled with FM4-64. NaCl was added to a final concentration of 0.5 M NaCl (t = 0 min) to induce vacuole fragmentation. During the following 12 min of incubation at 30 °C, the cells were analyzed by taking stacks of confocal images. Maximum-intensity projections of FM4-64 fluorescence in the stacks are shown, covering 6 μm in the z-direction. (**B**) Classification of the cells shown in panel A according to the number of vacuoles per cell. 200–400 cells were analyzed per strain (n = 3–5).

Due to this contradiction we explored the possibility that expression of GFP-AVP1 as such could impact vacuolar morphology in ways independent of vacuolar acidity, for example via vacuole fusion and fragmentation^2^,^3^,^6^,^30^,^34^,^51^,^52^,^53^,^54^. We challenged cells with 0.5 M NaCl, which induces rapid and synchronous fragmentation of wildtype vacuoles, following an ordered series of steps with different protein requirements^55^. In V-ATPase-proficient cells expressing GFP-AVP1 (SVY12), we could only analyze the part of the population that contained large vacuoles. Expression of GFP-AVP1 prevented the vacuoles in these cells from fragmenting upon salt treatment, and even from forming membrane invaginations that precede the actual fission event (Fig. 8A,B). This suggests that GFP-AVP1 interferes with vacuolar fragmentation. These observations indicate that the introduction of GFP-AVP1 itself has side effects on vacuolar structure and functionality that manifest themselves even in presence of an intact V-ATPase. Therefore, GFP-AVP1 is not a suitable tool to substitute for V-ATPase proton pump function in studies aiming at an analysis of vacuole fusion via the structure of the organelle *in vivo*.

## Discussion

Vacuole fusion can be assessed in different ways *in vivo*. Steady state vacuole structure has served as a readout because reductions in vacuolar fusion activity often coincide with a fragmentation of the vacuole *in vivo*^2^,^3^,^6^. We analyzed vacuole structure *in vivo* using time-lapse microscopy and acute inactivation of V-ATPase pump function by glucose withdrawal or by concanamycin A, a drug that blocks V-ATPase-dependent proton pumping but not vacuole fusion *in vitro* when used at appropriate low concentrations^9^,^56^. This allowed us to directly observe that loss of the vacuolar proton gradient induces vacuole fusion on the time scale of minutes. Since the reported experiments of Coonrod *et al*. had led these authors to the opposite conclusion, we revisited their experiments and performed additional controls on the assays used in their study.

Coonrod *et al*. judged the influence of the proton gradient on vacuole fusion via steady state vacuole morphology, relying on the *vph1*^*R735Q*^ substitution in V_0_ to abolish V-ATPase pump function. Unlike *vph1*^*R735Q*^, which shows strongly fragmented vacuoles, all V_1_ deletion mutants eliminate V-ATPase pump function but they maintain an intact V_0_ sector^31^,^35^ and do not show fragmented vacuoles. Instead, they tend to show a fusion of vacuoles into a single, large organelle. The same is true for wildtype cells in which V-ATPase pump activity has been acutely reduced by pharmacological inhibition or by physiological V_0_/V_1_ dissociation following glucose depletion. Thus, the typical result of suppression of V-ATPase-dependent proton pumping is vacuole fusion. Coonrod *et al*. assumed that the *vph1*^*R735Q*^ mutant left V_0_ structurally unchanged. This assumption may not be justified given that *vph1*^*R735Q*^ interferes with the physiological dissociation of the V_0_/V_1_ interaction that occurs upon glucose withdrawal from yeast cells^32^,^36^. Since R735 is located in the transmembrane region of Vph1 it cannot directly interact with any V_1_ subunit^57^,^58^,^59^. *vph1*^*R735Q*^ must hence influence V_1_–V_0_ dissociation indirectly, probably via an effect on V_0_ conformation. This interpretation is consistent with numerous observations indicating that V_0_ and its subunits can assume alternative conformations^7^,^37^,^38^,^56^,^58^,^60^,^61^. Therefore, the *vph1*^*R735Q*^ allele cannot be used to dissociate physical and proton pump-related roles of V_0_ in vacuole fusion in yeast.

Coonrod *et al*. also assayed vacuole fusion by mating cells. The vacuoles of the mating partners were labeled with different fluorophores and co-localization of these fluorophores in the vacuole of the first diploid daughter forming from the zygotes was taken as a readout for fusion. Co-localization was not observed in experiments with the *vph1*^*R735Q*^ mutant. Due to the constraints of this mutant that have been outlined above, these data cannot argue in favour of or against a physical or pump-related function of V_0_ in vacuole fusion. We were surprised to observe that, in contrast to cells lacking the vacuolar Q-SNARE (*vam3Δ*), cells lacking the cognate R-SNARE (*nyv1Δ*) did fuse their vacuoles in the daughter. This suggests that vacuoles in daughters emerging from zygotes fuse independently of Nyv1, although this protein promotes *in vivo* vacuole-vacuole fusion in other stages of the yeast life cycle, for example when haploid cells grow in the vegetative life cycle^1^,^51^,^62^. Nyv1 might be substituted for by another R-SNARE. A likely candidate is Ykt6, which binds to Vam3 and can replace Nyv1 under certain conditions *in vitro*^28^,^63^. We could not test the requirement for Ykt6 in the mating assay because Ykt6 is essential for cell growth and survival^64^.

Whereas microscopic analysis shows that vacuole fusion in the daughter of *nyv1Δ* zygotes proceeds as in wildtypes, assay of the same reaction via the maturation of pro-ALP by proteinase A had suggested that vacuole fusion was quantitatively blocked in *nyv1Δ* daughters^25^. We cannot provide a rational explanation how these authors could have observed Nyv1-dependent maturation of pro-ALP. These data are rendered even less plausible by the fact that, as we have shown above experimentally and theoretically, the ALP signals in this assay stem entirely from pro-ALP and proteinase A that have been produced in the diploid offspring and not before mating. Therefore, the pro-ALP maturation assay cannot report vacuole-vacuole fusion in daughter cells. It might at best measure effects on biosynthetic transport to the vacuole, but even then the reported effect of *nyv1Δ* cells remains unexplained because these biosynthetic transport routes are Nyv1-independent^47^.

An argument for the notion that vacuolar acidification was necessary and sufficient for vacuole fusion was also derived from the partial suppression of vacuolar fragmentation in *vph1Δ* mutants by expression of the heterologous pyrophosphate-driven proton pump AVP1^25^. This experiment cannot provide a compelling argument because, as our control experiments demonstrate, GFP-AVP1 itself severely distorts vacuolar morphology and also interferes with vacuole fission. This is critical because vacuole structure *in vivo* is determined by an equilibrium of fusion and fission activity, i.e. a diminution of fission activity can rescue vacuole fragmentation^51^,^52^,^53^,^65^. GFP-AVP1 produces these effects even in V-ATPase-proficient cells with acidified vacuoles, suggesting that GFP-AVP1 perturbs vacuole structure independently of a rescue of vacuolar acidification. We assume that GFP-AVP1 might perturb the metabolism of pyrophosphate, which is a key metabolic intermediate for many nucleotide-dependent reactions. This is underlined by the fact that deletion or downregulation of the yeast pyrophosphatase gene IPP1 is lethal^49^,^66^. Downregulation or deletion of IPP1 is, however, necessary in order to let pyrophosphate accumulate to levels high enough to fuel GFP-AVP1 and allow it to work as a proton pump^48^. These levels may be detrimental to the cells and affect vacuolar functionality and structure. Although we could not obtain the *ipp1Δ* strain and hence the strain background is not identical to the one used by Coonrod *et al*., our results nevertheless indicate that the effects of GFP-AVP1 on vacuole structure cannot be attributed to a rescue of vacuolar pH.

In sum, our experimental controls and theoretic considerations leave no valid argument supporting a positive role for vacuolar acidification in vacuole-vacuole fusion *in vivo*. By contrast, our *in vivo* results are in line with a series of *in vitro* studies on vacuole fusion which had demonstrated a physical requirement of V_0_ for fusion but which had failed to detect significant effects of pharmacological or genetic ablation of V-ATPase pump activity^1^,^7^,^9^,^15^,^51^,^52^,^56^. The results from our *in vivo* analysis of vacuole fusion are also supported by a series of studies in living cells from numerous other systems. In *Drosophila melanogaster*, the effect of R735 substitutions in the a subunit v100 had been analyzed^20^, an experiment that Coonrod *et al*. had proposed as a means to re-evaluate the role of acidification V_0_-dependent fusion reactions in non-yeast systems. The *v100*^*R755A*^ allele affects the same residue as the vph1^R735Q^ allele in yeast and it does not support acidification. However, *v100*^*R755A*^ does support fusion at the presynaptic membrane and on endosomes^20^. In *Caenorhabditis elegans*, secretion of multivesicular bodies is inhibited by point mutations of the V_0_ a-subunit that preserve proton pump function. Knockout of the catalytic V_1_ sector, which eliminates V-ATPase proton pumping, did not have this effect^18^, separating the secretion defect from V-ATPase pump function. In glia of zebrafish, phagosome-lysosome fusion is inhibited by ablation of the V_0_ a-subunit Atp6v0a1 although these organelles maintain their acidification^19^. In yeast, numerous point mutants in the V_0_ proteolipids support proton pump function while leading to significant vacuole fragmentation^15^,^41^,^42^ *in vivo*. Also covalent fusion of two proteolipid subunits of V_0_ by a peptide linker located at the lumenal face of the membrane (in Vma16-3HA), i.e. at the opposite side of where fusion is initiated, induced vacuole fragmentation *in vivo*. Also this construct supports proton pump function *in vivo* and *in vitro*^15^,^27^,^67^ but interferes with vacuole fusion *in vivo* that is triggered by an acute loss of proton pump activity. In chromaffin cells, rapid photo-inactivation of V_0_ subunits reduces the frequency of regulated exocytic events and the shape of the exocytic spikes, suggesting that V_0_ is in contact with the fusion pore. Acute ablation of V_1_ subunits by photo-inactivation or pharmacological inhibition of the V-ATPase did not have these effects^24^,^25^, indicating that they cannot be attributed to a loss of proton pump function. Finally, V_0_ proteolipids interact with SNAREs and this interaction is required for regulated exocytosis but not for proton pumping^22^.

In sum, a substantial body of work, stemming from *in vivo* studies in a variety of organisms and cell types and from *in vitro* studies on the fusion of vacuoles^7^,^9^,^15^,^16^, supports the notion that V_0_ contributes physically to membrane fusion and separates this activity from its role in proton pumping. Our direct microscopic observations of vacuoles *in vivo* are consistent with these findings and indicate that the proton pumping activity even counteracts vacuole fusion. However, while published studies collectively make a clear case for a physical and pump-independent role of V_0_ in membrane fusion^1^,^7^,^9^,^15^,^51^,^52^,^56^ we should not generally exclude effects of acidification. These can be observed for various trafficking reactions^68^ and they can even synergize with physical effects. The latter has been elegantly demonstrated by a recent study in chromaffin cells which proposed that V-ATPase disassembles when the lumen of secretory vesicles is well acidified^24^. This triggered V_0_/V_1_ dissociation should liberate V_0_ sectors to physically support the fusion process only in vesicles which are acidified and therefore can be expected to be loaded with transmitters. Thus, many interesting facets of V-ATPase function in membrane trafficking remain to be discovered and progress on this question will depend on the development of further approaches to manipulate the physical and pump-related functions of the V-ATPase at different stages of a trafficking reaction.

## Materials and Methods

### Strains and culture conditions

Culture media were either synthetic complete (SC) or yeast extract/peptone/dextrose (YPD; 2% glucose), buffered to pH 5.5 with 50 mM MES buffer. Cultures were shaken at 30 °C and 150 rpm. Strains carrying expression plasmids were grown on the corresponding SC dropout medium selecting for the auxotrophic marker. Strains and plasmids used in this study are listed in Tables 1 and 2, respectively. The SVY9 strain was generated as described^48^,^50^. The deletion mutant SVY13 was generated by replacing VPH1 with a nourseothricin (clonNat) cassette from plasmid pFA6a-natNT2^69^ using SVY9 as parental strain.

### Plasmid construction

pRS416-P_Vph1_-Vph1 carries a *VPH1* gene under the control of the endogenous promoter (the region 500 bp upstream of the ORF). The *VPH1* gene in the vector contains two silent mutations introducing a unique PacI site. vph1R735Q and vph1R735K alleles were produced by the Quick Change mutagenesis protocol using the primers: R735K, TCCTATTTAAAGTTATGGGCCTTATCATTGGC and GGCCCATAACTTTAAATAGGATGCAGTGTGCG; R735Q, TCCTATTTACAATTATGGGCCTTATCATTGGC and GGCCCATAATTGTAAATAGGATGCAGTGTGCG. The mutations were transferred into the non-mutagenized vector using PacI and ClaI restriction nucleases and the presence of the mutations was verified by sequencing. The Vph1-GFP fusion protein vector was generated by placing an EGFP cassette at the 3′ end of the Vph1 ORF.

### Mating Assay

Cells from the two mating types (BY4741 and BY4742) were transformed with a plasmid encoding either mCherry-ALP or GFP-ALP as described in Coonrod *et al*. Transformants were grown in SC-LEU pH 5.5. Crosses were set up by diluting each mating type to an OD of 0.2 and incubating the samples at 30 °C with gentle mixing. Microscopy pictures were recorded on a confocal spinning disk microscope as described below after 4–6 hours of incubation.

### Vacuolar pH measurements

Vacuolar pH was measured as described^25^,^40^. Cells were grown in SC or YPD medium buffered to pH 5.5 with 50 mM MES to an OD_600nm_ of 0.8–1.2. Cells were sedimented by centrifugation (3'000 × g, 2 min, 20 °C) and resuspended in 100 μl of the same medium containing 50 μM BCECF-AM (2′,7′.bis-(2-carboxyethyl)-5-(and-6)-carboxyfluoresceine acetoxymethyl ester, Molecular Probes). The suspension was incubated at 30 °C for 30 min. Cells were washed twice in the same medium and finally resuspended at an OD_600nm_ of 1.0. For end-point measurements, 50 μl of the cells were added to 150 μl of 50 mM MES buffer pH 5.5 in a black 96-well plate. At the same time, a calibration curve was recorded for each strain. To this purpose, 50 μl of the cell suspension was mixed with 50 μl of 50 mM MES buffer pH 5.5 and 100 μl of 2x calibration buffer (50 mM MES, 50 mM HEPES, 50 mM KCl, 50 mM NaCl, 0.2 M ammonium acetate, 10 mM NaN_3_, 10 mM 2-deoxyglucose, 50 μM FCCP) adjusted to pH values between 5.0 and 8.0. Fluorescence measurements were recorded in a Spectramax Gemini XS fluorescence plate reader (Molecular Devices) with excitation wavelengths of 450 and 490 nm and emission wavelength of 535 nm. The ratio of fluorescence intensities (490 nm/450 nm) was used to estimate the vacuolar pH by comparison with the calibration curves. For kinetic experiments, the labeled cell suspension was incubated with a V-ATPase inhibitor or DMSO at 30 °C. A calibration curve was obtained at the beginning and at the end of the experiment to ensure that the changes observed did not result from the ageing of the labeled suspension.

### Vacuole staining and microscopy

Yeast cultures were grown over night at 30 °C in YPD or SC media (buffered to pH 5.5 with 50 mM MES) to an OD_600nm_ of 0.2 to 1.2. Cells were harvested at 3000 × g, resuspended in 2–3 ml of fresh media at an OD_600nm_ of 0.1 to 0.2. FM4-64 was added from a 10 mM stock in DMSO to a final concentration of 10 μM and cells were incubated at 30 °C for 1 h. After washing two times with media, cells were shaken for 1.5 to 2.5 h at 30 °C in the same media as before, but without FM4-64.

For confocal imaging, we used a Perkin-Elmer UltraView Vox Confocal Spinning Disk Setup on an inverted Zeiss Microscope with a 100x oil immersion objective (NA 1.41) and two Hamamatsu ORCA-Flash 4.0 cameras. FM4-64 was visualized in fast acquisition mode with excitation by the 488 nm laser. For stack imaging, 9 focal planes with a distance of 0.5 μm were acquired at each time point. Non-confocal microscopy was performed on an inverted Leica DMI6000B fluorescence microscope equipped with a 100x/1.4 NA lens and a Hamamatsu C10600-10B camera (ORCA-R2). Images were processed using ImageJ. Stack projections were obtained using the maximum intensity function. Brightness and contrast were linearly adjusted. Exposure of the cells to excitation light was kept at a minimum in order to avoid bleaching and light-induced fusion of vacuoles, which can occur upon prolonged illumination.

### Cell immobilization for live-microscopy of yeast cells

Lab-Tek 8-well chamber slides (Nunc, 155411) were coated with 35 μl of a 1 mg/ml aqueous solution of concanavalin (freshly prepared from a 10x frozen stock). After air drying the slides, FM4-64 stained cultures were harvested for 3 min at 3000 × g, most of the supernatant was removed, and cells were resuspended in 50–100 μl of the remaining medium. 25 μl of cells were pipetted into each well of the coated chamber slide, and allowed to settle for 5 to 10 min at 25 °C. After 3 washes with 400 μl medium, 200 μl medium was added and cells were transferred to the microscope.

Alternatively, cells were immobilized on Sticky-Slide VI slides (from Ibidi) after coating of the coverslip with concanavalin A as described above. Cells were grown in YPD pH 5.5 and perfused at a flow rate of 10 μl/min with intermittent breaks to allow binding of the cells. Then, cells were kept under constant flow of medium at 20 μl/min. Then, the medium was changed to either YPD pH 5.5 (Glc-Glc) or YPGal pH 5.5 (Glc-Gal). After 10 min under the same flow, image acquisition was started using the confocal microscope set up described above. The 488 nm laser was used at minimal power (7%) and acquisition time was kept low (30 ms) to minimize light-induced damage of the cells. A picture was recorded every 12 seconds.

### Glucose deprivation

Cells were grown at 30 °C over night to an OD_600nm_ = 0.2–1.2 in buffered SC media with 2% glucose. Cultures were stained with FM4-64 and chased as described above. Cultures were divided into three 500 μl aliquots, cells were harvested at 5000 rpm for 10 s in a micro-centrifuge, washed twice, and resuspended in SC medium containing 2% glucose, no glucose, or 2% galactose instead of glucose. Cells were harvested as before, washed once in the respective media, resuspended in 500 μl media, and immediately observed under the microscope. Samples were further incubated at 30 °C and analyzed by microscopy.

### Quantification of vacuole morphology

Vacuole morphology was quantified by counting cells in each indicated category from images taken with Perkin-Elmer UltraView Vox Confocal Spinning Disk or Zeiss Axioplan fluorescence microscope. Numbers of cells in each category are shown as a percentage of the total number of cells counted, and the SEM is indicated.

## Additional Information

**How to cite this article**: Desfougères, Y. *et al*. Organelle acidification negatively regulates vacuole membrane fusion *in vivo*. *Sci. Rep*. **6**, 29045; doi: 10.1038/srep29045 (2016).

## Supplementary Material

Supplementary Information

Supplementary Movie S1

Supplementary Movie S2

## Figures and Tables

**Table 1:** List of strains.

Strain	Genotype	Source
BY4741	MATa his3Δ1 leu2Δ0 met15Δ0 ura3Δ0	Euroscarf
BY4742	MATα his3Δ1 leu2Δ0 lys2Δ0 ura3Δ0	Euroscarf
BY4242 *vma1Δ*	*vma1Δ*::kanMX4	Euroscarf
BY4741 *vph1Δ*	*vph1Δ*::kanMX4	Euroscarf
BY4742 *vph1∆*	*vph1Δ*::kanMX4	Euroscarf
BY4741 *vam3Δ*	*vam3Δ*::kanMX4	Euroscarf
BY4742 *vam3Δ*	*vam3Δ*::kanMX4	Euroscarf
BY4741 *vma1Δ*	*vma1Δ*::kanMX4	Euroscarf
BY4741 *vma2Δ*	*vma2Δ*::kanMX4	Euroscarf
BY4741 *vma8Δ*	*vma8Δ*::kanMX4	Euroscarf
BY4741 *VPH1-R735Q*	BY4741 *vph1Δ* pRS416-Vph1R735Q (URA3)	This study
BY4741 *VPH1-R735K*	BY4741 *vph1Δ* pRS416-Vph1R735K (URA3)	This study
BY4741 16-3HA	*vma16Δ*::natNT2 *pep4Δ*::kanMX4 pRS316-Vma16-Vma3 (URA3)	Strasser *et al*.^15^
BY4741 Vma5-GFP	BY4741 Vma5-GFP (HIS3)	This study
SVY9	BY4741 ipp1::P_GAL1_-IPP1-TRP1	This study
SVY14	BY4741, pTcGFP-AVP1 (URA3)	This study
SVY12 (p:AVP1)	SVY9, pTcGFP-AVP1 (URA3)	This study
SVY13(*vph1Δ*+ p:AVP1)	SVY12, *vph1Δ*::natNT2	This study
BY4741 *nyv1∆*	*nyv1Δ*::kanMX4	Euroscarf
BY4742 *nyv1∆*	*nyv1Δ*::kanMX4	Euroscarf
KEBY136	*MATα ura3-52 leu2-3 112 his4-519 ade6 gal2 pep4-3 pho8∆-X*	Coonrod *et al*.^25^
KEBY136 CUP1-PHO8	*KEBY136 pTC2 (CUP1-PHO8)*	Coonrod *et al*.^25^

**Table 2:** List of plasmids.

Plasmid	Description	Source
pLG230	Yeast expression plasmid (CEN, LEU2) encoding GFP-ALP from the CPY promoter	Coonrod *et al*.^25^
pMP2	Yeast expression plasmid (CEN, LEU2) encoding mCherry-ALP from the VPH1 promoter with ALP terminator	Coonrod *et al*.^25^
Vph1^wt^	pRS416 Vph1	This study
Vph1^R735Q^	pRS416 Vph1R735Q	This study
Vph1^R735K^	pRS416 Vph1R735K	This study
Vph1-GFP	pRS416 Vph1-GFP	This study
Vma16-3HA (pLG146)	pRS316 PromVMA3 VMA16::3xHA::VMA3	Flannery *et al*.^26^
pTcGFP-AVP1	GFP-AVP1	Perez-Castineira *et al*.^48^
pTC2	Yeast expression plasmid (CEN, URA3) encoding PHO8 (alkaline phosphatase; ALP) from the CUP1 promoter	Coonrod *et al*.^25^
