# Supplementary material for: Organelle acidification negatively regulates vacuole membrane fusion in vivo
Source: Sci Rep. 2016 Jul 1;6:29045. doi: 10.1038/srep29045 (PMC4929563; doi:10.1038/srep29045)
Supplement: Supplementary Information [file srep29045-s1.pdf]

Organelle acidification negatively regulates vacuole membrane fusion in vivo

Yann Desfougères, Stefano Vavassori, Maria Rompf, Ruta Gerasimaite and Andreas Mayer

Supplementary Figures

Supplementary Figure 1: Localization of subunit a (Vph1-GFP) in *vma1Δ* cells.

Supplementary Figure 2: Entire field of SVY13 cells used in Fig. 7

Supplementary Figure 3: Entire field of SVY12 cells used in Fig. 7

Supplementary Figure 4: Entire field of SVY14 cells used in Fig. 7

Videos

Movie 1: Stable vacuole structure in medium with glucose

BY4741 cells stained with FM4-64 were fixed in microfluidic chambers. After immobilization, YPD was constantly flushed at a rate of 20 µl/min. Stacks were recorded every 12 seconds for 20 min. Acquisition was started only 10 min after the cells were attached to the slides. Stacks are shown at 5 frames per second.

Movie 2 : Vacuole fusion induced by shift from glucose to galactose.

BY4741 cells stained with FM4-64 were fixed in microfluidic chambers. After immobilization, YPD was constantly flushed at a rate of 20 µl/min. After the cells were attached to the slides, YPD was replaced by YPGal. Stacks were recorded every 12 seconds for 20 min. Since glucose depletion takes several minutes in order to become effective, recording was started only 10 min after the carbon source was switched to galactose in order to reduce bleaching and potential phototoxicity. Stacks are shown at 5 frames per second

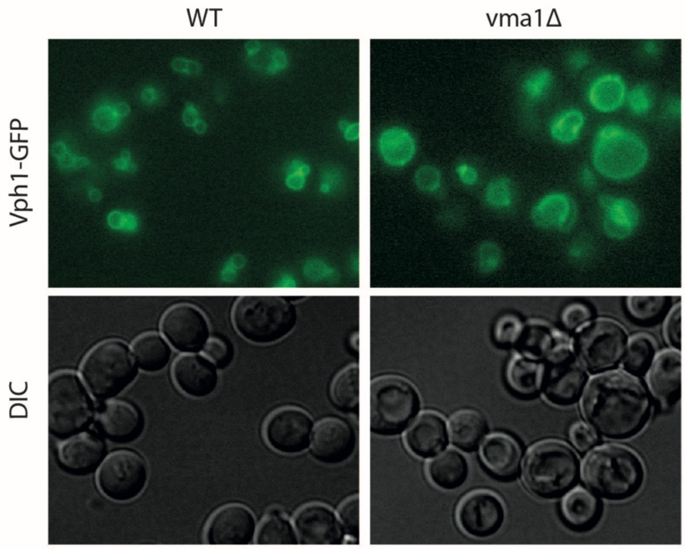

Supplementary Figure 1: Localization of subunit a (Vph1-GFP) in vma1Δcells.

Vph1-GFP was expressed from a plasmid in wildtype (BY4742) or isogenic vma1Δ cells that lack the V1 subunit A. Cells were grown logarithmically in YPD buffered to pH 5.5, harvested at OD600nm=1 and analyzed on a standard fluorescence microscope. The microscope settings for acquisition were identical for the two strains.

Desfougères et al., Suppl. Fig. 1

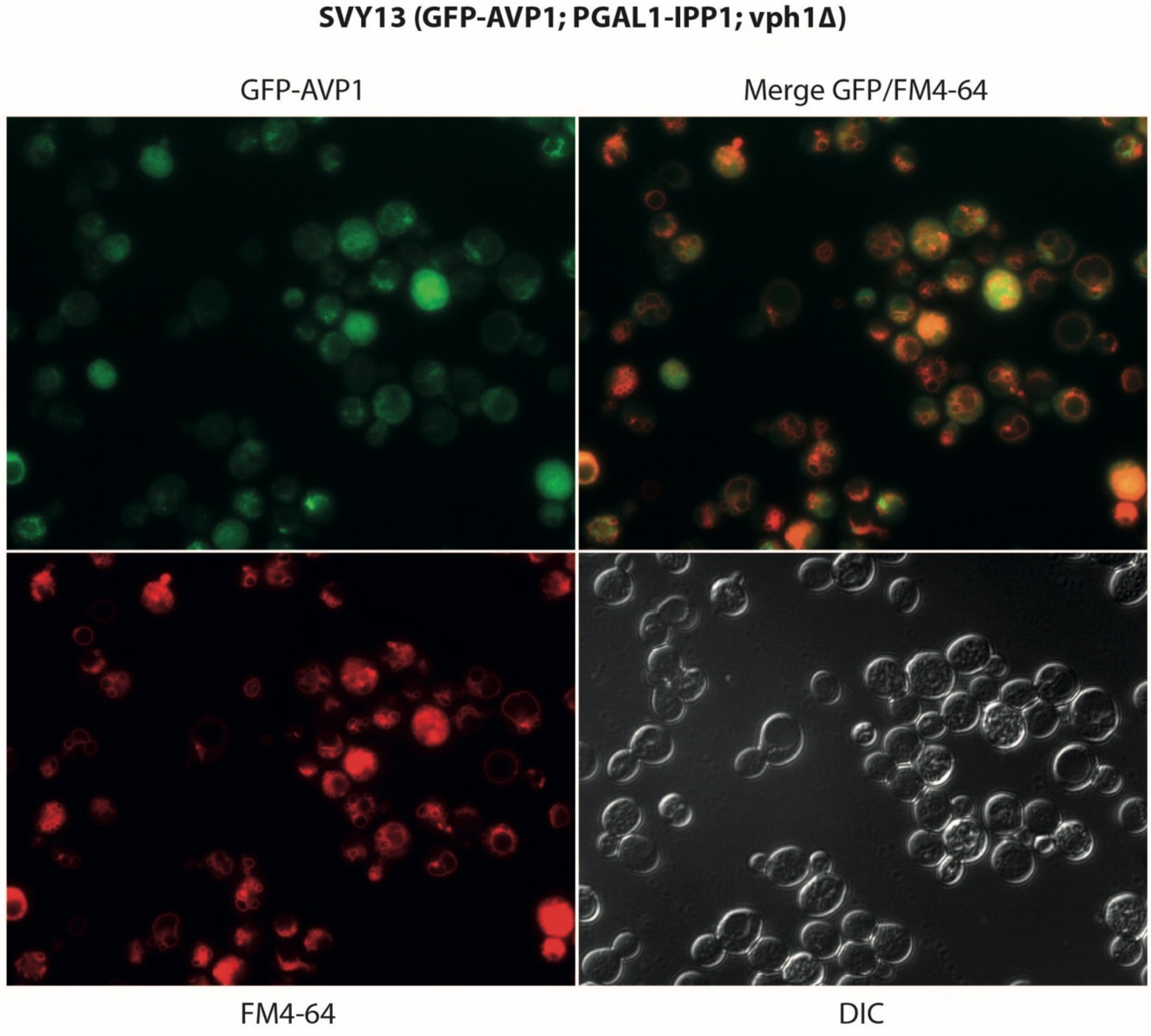

Supplementary Figure 2: Entire field of SVY13 cells used in Fig. 7

SVY12 (GFP-AVP1; PGAL1-IPP1)

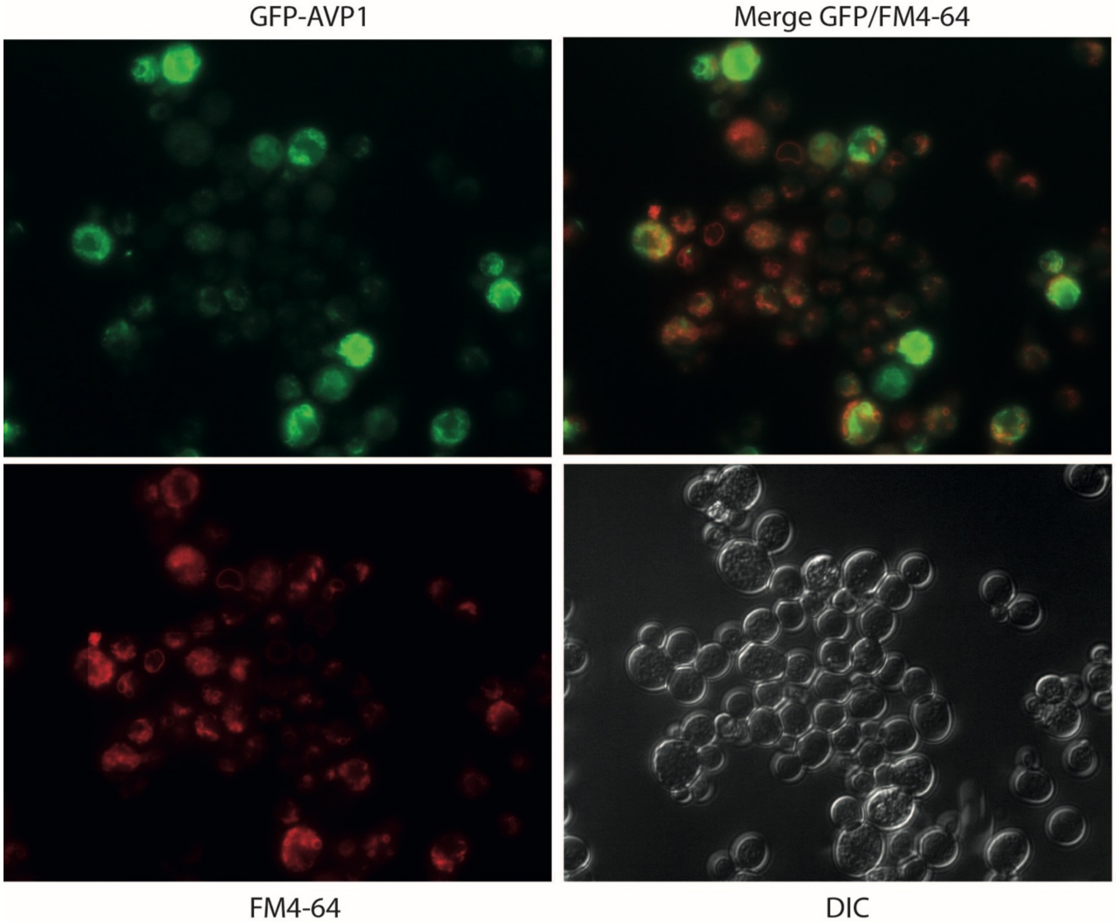

Supplementary Figure 3: Entire field of SVY12 cells used in Fig. 7

Desfougères et al., Suppl. Fig. 3

SVY14 (GFP-AVP1 only)

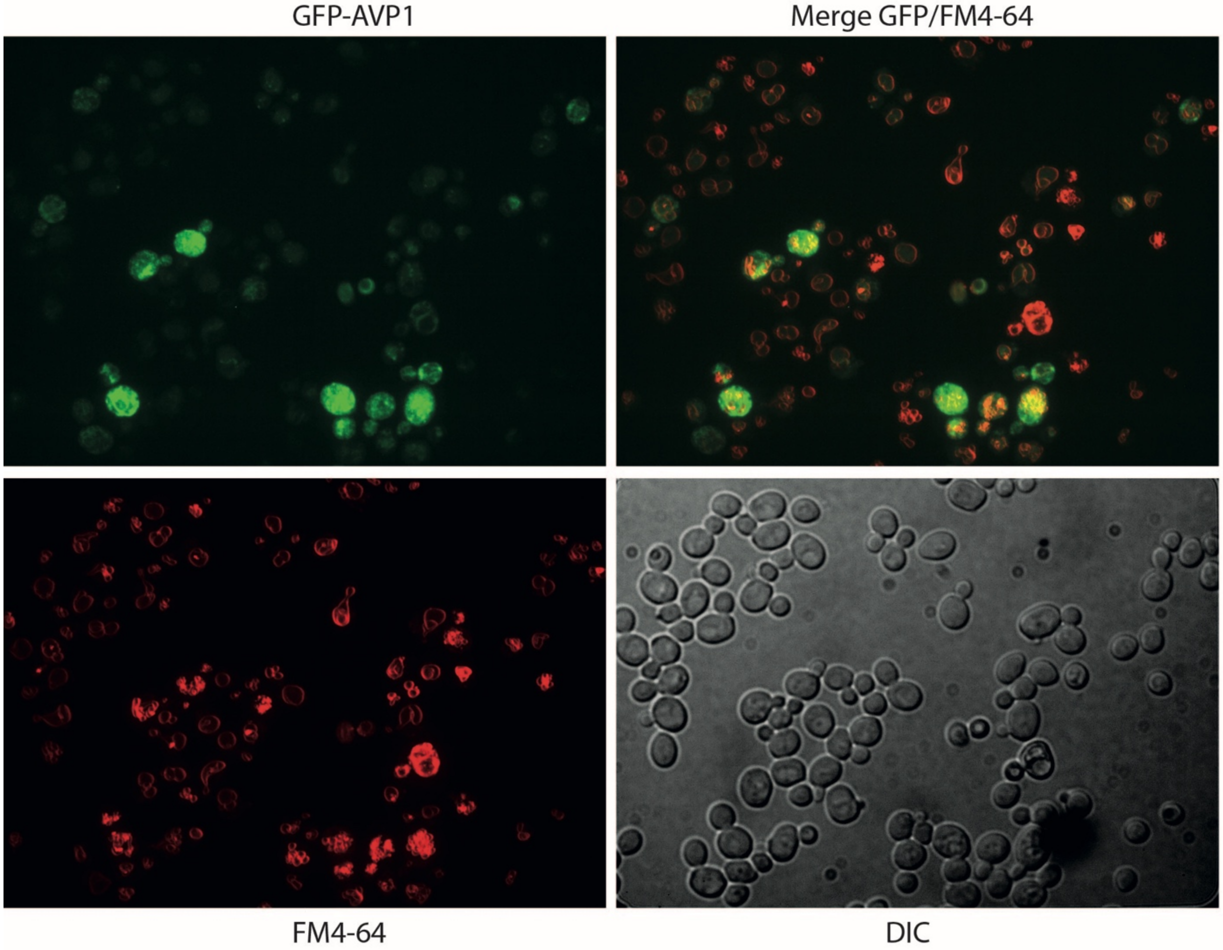

Supplementary Figure 4: Entire field of SVY14 cells used in Fig. 7

Desfougères et al., Suppl. Fig. 4
